# Association between *FAS* gene −670 A/G and −1377 G/A polymorphisms and the risk of autoimmune diseases: a meta-analysis

**DOI:** 10.1042/BSR20191197

**Published:** 2020-01-06

**Authors:** Hongwei Yan, Yuxiao Hong, Yunfei Cai

**Affiliations:** NHC/Ministry of Education/Liaoning Province Key Laboratory of Immunodermatology (China Medical University), Department of Dermatology, The First Hospital of China Medical University, No.155 Nanjing Bei Street, Heping District, Shenyang, Liaoning Province, P.R. China 110001

**Keywords:** Autoimmune Diseases, FAS, Meta-analysis, polymorphisms

## Abstract

**Objectives:** FAS plays a critical role in the extrinsic apoptosis pathway in autoimmune diseases. Previous studies investigating the association between *FAS* gene −670 A/G and −1377 G/A polymorphisms and the risk of autoimmune diseases reported controversial results. We performed the meta-analysis to evaluate the possible association. **Methods:** Relevant studies were identified by searching the PubMed, Embase, CNKI, and Wanfang databases up to December 2018. Odds ratios (ORs) and corresponding 95% confidence intervals (CIs) were calculated to determine the association. **Results:** A total of 43 articles including 67 studies (52 studies for *FAS* −670 A/G and 15 studies for −1377 G/A) were included in the meta-analysis. Our meta-analysis showed that the *FAS* −670 A/G polymorphism was associated with the risk of autoimmune diseases (GG vs. GA: OR = 1.079, 95% CI = 1.004–1.160, *P*=0.038), especially in Caucasians (GG vs. GA: OR = 1.12, 95% CI = 1.03–1.23, *P*=0.012), Asians (G vs. A: OR = 0.89, 95% CI = 0.83–0.96, *P*=0.002), systemic lupus erythematosus (SLE) (G vs. A: OR = 0.85, 95% CI = 0.77–0.94, *P*=0.001), multiple sclerosis (MS) (GG+GA vs. AA: OR = 0.83, 95% CI = 0.70–0.99, *P*=0.043), systemic sclerosis (SSc) (GG vs. GA: OR = 1.20, 95% CI = 1.07–1.36, *P*=0.003) and Hashimoto’s thyroiditis (HT) (G vs. A: OR = 1.45, 95% CI = 1.10–1.90, *P*=0.008); the *FAS* −1377 G/A polymorphism was associated with the risk of autoimmune diseases (A vs. G: OR = 1.11, 95% CI = 1.03–1.20, *P*=0.008), especially in Asians (A vs. G: OR = 1.15, 95% CI = 1.05–1.25, *P*=0.002) and high quality studies (A vs. G: OR = 1.14, 95% CI = 1.05–1.24, *P*=0.002). **Conclusion:** This meta-analysis demonstrated that the *FAS* –670A/G and –1377 G/A polymorphisms were associated with the risk of autoimmune diseases.

## Introduction

Autoimmune diseases are chronic disorders characterized by the loss of immune tolerance to self-antigens, leading to immune-mediated tissue destruction. They affect 4–5% of adults, the majority of whom are women [1]. Co-occurrence of distinct autoimmune diseases within a single family and genome-wide association studies (GWASs) support the hypothesis that these diseases share common genetic risk factors [2–6]. The etiology of autoimmune diseases is attributed to complex interactions of genetics, epigenetics, and environmental factors that remain to be elucidated [7–12].

FAS (also known as APO-1, CD95, or TNFSF6) is a cell surface receptor that belongs to the tumor necrosis factor (TNF) receptor superfamily [13]. FAS is widely expressed in normal human tissues. To maintain self-tolerance, the binding of FAS-ligand (FASL) to FAS on the cell surface initiates the extrinsic apoptosis pathway [14]; thus, autoreactive lymphocytes are normally eliminated. However, abnormal apoptosis may lead to a failure to eliminate autoreactive lymphocytes, which can induce the appearance and development of autoimmune diseases [15]. The *FAS* gene is located on chromosome 10q24.1 in humans and is highly polymorphic [16]. In some individuals, there is an A to G substitution at position 670 and a G to A substitution at position 1377 in the *FAS* promoter region [17]. The *FAS* −670 A/G and −1377 G/A polymorphisms may destroy signal transducer and activator of transcription protein 1 (STAT1) and stimulatory protein 1 (SP1) transcription factor binding sites, resulting in reduced promoter activity and *FAS* expression [18]. Abnormal apoptosis mediated by the FASL interaction with the FAS receptor is involved in the pathogenesis of several autoimmune diseases and cancers [19].

Many studies have investigated the relationship between the *FAS* −670 A/G rs1800682 and −1377 G/A rs2234767 polymorphisms and the risk of autoimmune diseases [15,17,20–60], including systemic lupus erythematosus (SLE), rheumatoid arthritis (RA), multiple sclerosis (MS), autoimmune hepatitis (AIH), alopecia areata (AA), lupus nephritis (LN), systemic sclerosis (SSc), primary Sjögren’s syndrome (pSS), Hashimoto’s thyroiditis (HT), Guillain–Barré syndrome (GBS), primary biliary cirrhosis (PBC), vitiligo, Graves’ disease (GD), type 1 diabetes mellitus (T1D), idiopathic aplastic anemia (IAA), juvenile idiopathic arthritis (JIA), and spondyloarthropathies (SPA). However, previous results have been controversial, perhaps due to small sample sizes and low statistical power. Meta-analysis could provide more reliable results, enabling the inclusion of a larger sample size and enhanced statistical power by combining the results of independent eligible studies. Seven previous meta-analyses [43,61–66] have analyzed the association between the *FAS* −670 A/G or −1377 G/A polymorphisms and some autoimmune diseases. However, these studies only analyzed SLE, RA, LN, SSc, pSS, JIA, SPA, and AIH and did not include all autoimmune diseases. Furthermore, previous meta-analyses [63,65] including several studies [25,30,31,40] contained some errors when extracting the data. Thus, in the present study, we aimed to perform a meta-analysis to investigate whether the *FAS* −670 A/G or −1377 G/A polymorphisms is associated with autoimmune diseases risk by including 23 new articles, consisting of 33 studies [15,17,22,27–30,32–35,37,41,43–45,50,52–55,59,60] on SLE, MS, pSS, AA, PBC, HT, GBS, LN, vitiligo, T1D, IAA, and GD and correcting the errors in the previous meta-analyses. To our knowledge, this is the most comprehensive meta-analysis to assess the association of an *FAS* polymorphisms with the risk of autoimmune diseases, including SLE, RA, MS, AIH, LN, SSc, AA, pSS, HT, GBS, PBC, vitiligo, GD, T1D, IAA, JIA, and SPA.

## Methods

This meta-analysis was conducted and reported according to the Preferred Reporting Items for Systematic Reviews and Meta-Analyses (PRISMA) 2009 checklist [67].

### Literature search

Literature published in English and Chinese was retrieved from the PubMed, Embase, CNKI, and Wanfang databases up to December 2018. The search strategy used the following medical subject heading (MeSH) terms combined with text words: ‘FAS or TNFRSF6 or CD95 or APO-1 or rs1800682 or rs2234767’, ‘polymorphism, genetic or polymorphisms or polymorphism or variant or mutation’ and ‘autoimmune diseases or autoimmune disease or autoimmunity’. A manual search of the reference lists was also performed to identify additional articles.

### Inclusion and exclusion criteria

Studies meeting all the following criteria were included in the analysis: (1) evaluation of the association between the *FAS* −670 A/G or −1377 G/A polymorphisms and autoimmune diseases risk; (2) available and sufficient genotype data to calculate the odds ratio (OR) with 95% confidence interval (CI); and (3) a case–control study design.

Studies were excluded if they met the following criteria: (1) containing overlapping data; (2) not containing genotype data from the cases and controls; and (3) reviews, case reports, abstracts, letters, animal experiments and meta-analyses.

### Data extraction

Two investigators independently assessed and extracted data from all included studies. Discrepancies were resolved by discussion. The following data were collected from each study: disease type, first author, year of publication, country, ethnicity, genotyping method, sample sizes of cases and controls, genotype frequencies in cases and controls, and *P*-value of test for Hardy–Weinberg equilibrium (HWE) in controls.

### Quality evaluation

The methodological quality of the included studies was assessed independently by two investigators using the Newcastle–Ottawa scale (NOS) score [68]. The NOS score ranges from 0 to 9 and encompasses three components, including selection, comparability, and exposure. A study with score greater than or equal to 6 was considered of high methodological quality. Discrepancies were resolved by discussion.

### Statistical analysis

The chi-square test was applied to examine whether the observed genotype frequencies in controls conformed to HWE, and *P*<0.05 was considered to deviate from HWE. The ORs with their 95% CIs were used to assess the strength of associations between the *FAS* −670 A/G and −1377 G/A polymorphisms and autoimmune diseases. The statistical significance of the pooled ORs was determined by the *Z* test. The allelic (*FAS* −670 A/G: G vs. A; *FAS* −1377 G/A: A vs. G), homozygous (*FAS* −670 A/G: GG vs. AA; *FAS* −1377 G/A: AA vs. GG), heterozygous (*FAS* −670 A/G: GG vs. GA; *FAS* −1377 G/A: AA vs. AG), dominant (*FAS* −670 A/G: GG + GA vs. AA; *FAS* −1377 G/A: AA+AG vs. GG), and recessive (*FAS* −670 A/G: GG vs. GA+ AA; *FAS* −1377 G/A: AA vs. AG+GG) models were examined. The between-studies heterogeneity was assessed by *Q* test and quantified by *I*^*2*^ test [69]. When *P*≥0.1 or *I^2^* < 50%, there was no heterogeneity, and pooled OR estimates were combined using the fixed-effects model (Mantel–Haenszel method); otherwise, the random-effects model (Mantel–Haenszel method) was used to combine summary data [70]. To detect the main sources of heterogeneity, subgroup analyses were performed by ethnicity, disease type and quality score. Sensitivity analysis was carried out by excluding studies deviating from HWE to assess the stability of the meta-analysis. Egger’s test was used to assess publication bias [71]. If there was publication bias, we recalculated the adjusted ORs using the trim-and-fill method [72] to evaluate the possible impact of publication bias. The trim-and-fill method was used to impute hypothetical missing studies. For significant results observed in the current meta-analysis, the false-positive report probability (FPRP) test was utilized to examine positive associations. An FPRP threshold of 0.5 and a prior probability of 0.1 were set to detect an OR of 0.67/1.50 (protective/risk effects) for an association with the tested genotypes. FPRP values less than 0.5 were considered as noteworthy associations [73]. All statistical analyses were conducted using Stata 15 software (Stata Corporation, College Station, TX, U.S.A.). Results with *P*<0.05 were considered significant.

### Trial sequential analysis

Traditional meta-analysis may yield type I errors due to dispersed data or repetitive significance testing when new studies are added to it [74,75]. Trial sequential analysis (TSA) was used to minimize the risk of type I errors by calculating required information size (RIS) (meta-analysis sample size) and adjusted threshold for statistical significance [76]. TSA was performed by using TSA software 0.9.5.10 Beta (http://www.ctu.dk/tsa/) in the allelic model with the overall included studies by setting an overall type I error of 5%, power of 80%, relative risk reduction (RRR) of 20%, and control event proportion [77]. If the cumulative Z-curve crosses the trial sequential monitoring boundary or the RIS line, a reliable and conclusive evidence has been reached and further studies are not needed. Otherwise, more studies are needed to reach a firm conclusion.

## Results

### Characteristics of the included studies

**Figure 1:**
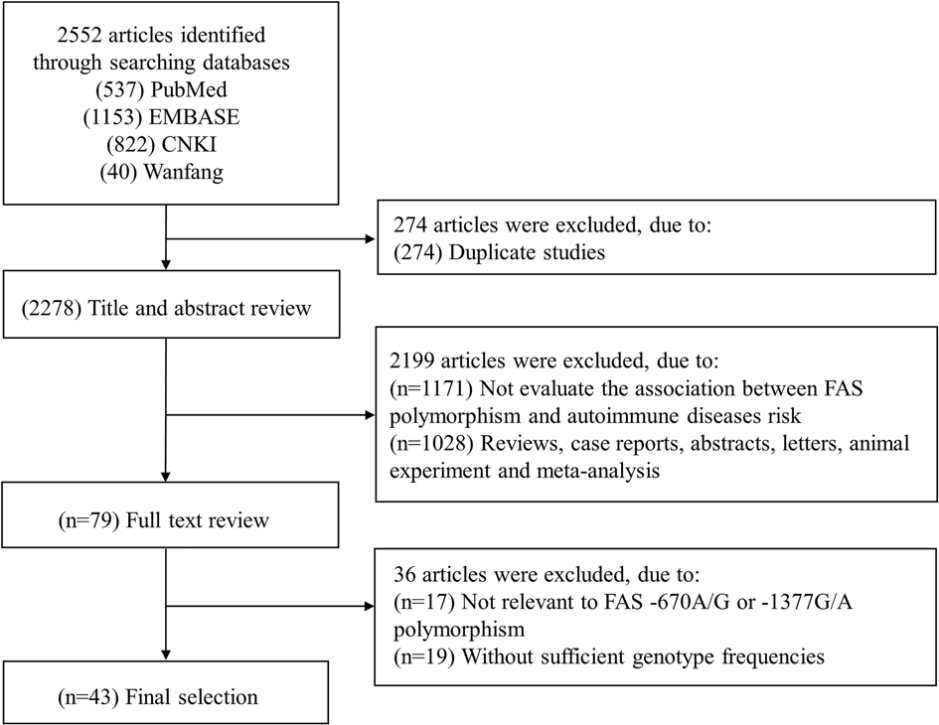
Flow diagram of the study selection process

A flowchart of the selection of eligible articles is presented in Figure 1. The initial search identified 2552 articles through the search strategy, and a total of 43 articles [15,17,20–60], consisting of 67 studies comprising 13340 patients and 14547 controls, were finally included in the meta-analysis according to the inclusion and exclusion criteria. Fifty-two studies examined the *FAS* −670 A/G polymorphism, and 15 studies examined the *FAS* −1377 G/A polymorphism. The characteristics of the articles included in the meta-analysis are summarized in Table 1.

**Table 1:** Characteristics of the case–control studies association of *FAS* −670A/G and −1377G/A polymorphisms and autoimmune diseases

Disease type	Polymorphism	Author	Year	Country	Ethnicity	Genotyping method	Sample size (case/control)	Case (GG/GA/AA)			Control (GG/GA/AA)			HWE	NOS
											*P*-value	score			
SLE	FAS −670A/G	Bollain et al.	2014	Mexico	Mestizos	PCR-RFLP	43/54	16	13	14	14	12	28	<0.001	5
		Moudi et al.	2013	Iran	Caucasian	PCR-RFLP	106/149	17	55	34	39	73	37	0.808	7
		Molin et al.	2012	Germany	Caucasian	PCR	46/96	8	21	17	34	51	11	0.213	4
		Lu et al.	2012	China	Asian	PCR	552/718	96	237	219	138	326	254	0.070	6
		Pradhan et al.	2012	India	Indian	PCR-RFLP	70/70	11	37	22	21	42	7	0.036	6
		Arasteh et al.	2010	Iran	Caucasian	ASO-PCR	249/212	74	93	82	58	98	56	0.273	7
		Xu et al.	2004	China	Asian	PCR-RFLP	103/110	15	59	29	23	61	26	0.249	5
		Kanemitsu et al.	2002	Japan	Asian	AS-PCR, PCR-SSCP	109/140	25	49	35	50	64	26	0.492	5
		Lee et al.	2001	Korea	Asian	PCR-RFLP	87/87	13	47	27	13	48	26	0.230	5
		Huang et al.	1999	Australia	Caucasian	PCR-RFLP	79/86	20	21	38	20	22	44	<0.001	4
MS	FAS −670A/G	Mohammadzadeh et al.	2012	Iran	Caucasian	PCR-RFLP	107/112	22	37	48	18	50	44	0.551	8
		Kantarci et al.	2004	U.S.A.	Caucasian	PCR-RFLP	218/441	37	108	73	86	234	121	0.154	8
		Lucas et al.	2004	Spain	Caucasian	PCR	320/218	68	177	75	44	113	61	0.525	7
		Niino et al.	2002	Japan	Asian	PCR-RFLP	114/121	23	65	26	25	63	33	0.614	7
		van Veen et al.	2002	Netherlands	Caucasian	PCR	383/206	80	185	118	42	118	46	0.036	6
		Huang et al.	2000	Australia	Caucasian	PCR-RFLP	124/183	22	58	44	40	97	46	0.407	7
RA	FAS −670A/G	Yıldır et al.	2013	Turkey	Caucasian	TaqMan	100/101	20	45	35	22	40	39	0.063	7
		Kobak et al.	2012	Turkey	Caucasian	PCR-RFLP	101/105	24	50	27	14	52	39	0.608	5
		Mohammadzadeh et al.	2011	Iran	Caucasian	PCR	120/112	17	64	39	18	50	44	0.551	4
		Lee et al.	2001	Korea	Asian	PCR-RFLP	87/87	16	38	33	13	48	26	0.230	5
		Huang et al.	1999	Australia	Caucasian	PCR-RFLP	185/86	32	105	48	22	44	20	0.825	4
		Coakley et al.	1999	U.S.A.	Caucasian	PCR	18/128	4	8	6	31	61	36	0.607	4
AIH	FAS −670A/G	Ngu et al.	2013	New Zealand	Caucasian	PCR	77/455	19	35	23	107	214	134	0.232	5
		Su et al.	2012	China	Asian	PCR-RFLP	48/68	5	24	19	20	30	18	0.335	6
		Agarwal et al.	2007	U.S.A.	Caucasian	PCR	149/172	35	75	39	32	84	56	0.960	4
		Hiraide et al.	2005	Japan	Asian	PCR	72/130	14	31	27	40	63	27	0.811	4
LN	FAS −670A/G	Bollain et al.	2014	Mexico	Mestizos	PCR-RFLP	24/54	8	9	7	14	12	28	<0.001	5
		Pradhan et al.	2012	India	Indian	PCR-RFLP	35/70	7	16	12	21	42	7	0.036	6
		Xu et al.	2004	China	Asian	PCR-RFLP	62/110	9	34	19	23	61	26	0.249	5
		Lee et al.	2001	Korea	Asian	PCR-RFLP	26/87	4	12	10	13	48	26	0.230	5
SSc	FAS −670A/G	Liakouli et al.	2013	Italy	Caucasian	PCR	350/232	65	158	127	60	120	52	0.586	8
		Broen et al.	2009	Europe, U.S.A.	Caucasian	TaqMan	2565/2855	616	1205	744	586	1455	814	0.168	7
		Broen et al.	2009	U.S.A.	Hispanic	TaqMan	159/137	46	80	33	41	71	25	0.552	7
		Broen et al.	2009	U.S.A.	African	TaqMan	176/194	93	68	15	96	83	15	0.613	7
AA	FAS −670A/G	Seleit et al.	2018	Egypt	Caucasian	PCR	60/40	14	37	9	4	23	13	0.181	8
		Kalkan et al.	2013	Turkey	Caucasian	PCR-RFLP	118/118	0	81	37	13	65	40	0.077	7
		Fan et al.	2010	China	Asian	PCR	84/84	13	35	36	13	49	22	0.099	6
pSS	FAS −670A/G	Treviño-Talavera et al.	2014	Mexico	Amerindian	PCR-RFLP	77/84	25	32	20	22	42	20	0.996	4
		Mullighan et al.	2004	Australia	Caucasian	PCR	101/108	17	54	30	21	54	33	0.897	4
		Bolstad et al.	2000	Norway	Caucasian	PCR	70/72	26	26	18	12	39	21	0.394	5
HT	FAS −670A/G	Erdogan et al.	2016	Turkey	Caucasian	PCR-RFLP	112/112	31	57	24	15	56	41	0.547	8
		Inoue et al.	2016	Japan	Asian	PCR-RFLP	117/80	33	53	31	20	37	23	0.510	6
GBS	FAS −670A/G	Islam et al.	2018	Japan	Asian	PCR	300/300	51	114	135	45	126	129	0.125	7
		Geleijns et al.	2005	Netherlands	Caucasian	PCR	272/212	67	129	76	42	114	56	0.243	5
PBC	FAS −670A/G	Su et al.	2012	China	Asian	PCR-RFLP	19/68	5	7	7	20	30	18	0.335	6
		Hiraide et al.	2005	Japan	Asian	PCR	96/130	30	37	29	40	63	27	0.811	4
vitiligo	FAS −670A/G	Li et al.	2008	China	Asian	PCR	750/756	101	364	285	108	363	285	0.660	7
GD	FAS −670A/G	Inoue et al.	2016	Japan	Asian	PCR-RFLP	146/80	41	61	44	20	37	23	0.510	6
T1D	FAS −670A/G	Sahin et al.	2012	Turkey	Caucasian	PCR	85/80	13	46	26	10	40	30	0.551	7
IAA	FAS −670A/G	Rehman et al.	2018	Pakistan	Caucasian	PCR	170/222	13	105	52	26	47	149	<0.001	7
JIA	FAS −670A/G	Donn et al.	2002	U.K.	Caucasian	PCR-RFLP	342/255	79	177	86	48	139	68	0.122	4
SPA	FAS −670A/G	Lee et al.	2001	Korea	Asian	PCR	54/84	11	27	16	13	46	25	0.279	5
								Case (AA/AG/GG)			Control (AA/AG/GG)				
SLE	FAS −1377A/G	Arasteh et al.	2010	Iran	Caucasian	ASO-PCR	249/212	3	43	203	6	54	152	0.652	7
		Kanemitsu et al.	2002	Japan	Asian	AS-PCR, PCR-SSCP	109/140	25	42	42	33	62	45	0.202	5
		Huang et al.	2000	Australia	Caucasian	PCR	86/90	3	21	62	2	22	66	0.917	7
RA	FAS −1377A/G	Zhu et al.	2016	China	Asian	MALDI-TOFMS	615/839	68	284	246	85	357	389	0.817	7
		Yıldır et al.	2013	Turkey	Caucasian	TaqMan	100/101	0	26	74	2	18	81	0.411	7
pSS	FAS −1377A/G	Mullighan et al.	2004	Australia	Caucasian	PCR	101/108	4	14	83	1	19	88	0.982	4
		Bolstad et al.	2000	Norway	Caucasian	PCR	70/72	2	18	50	1	18	53	0.702	5
GBS	FAS −1377A/G	Islam et al.	2018	Japan	Asian	PCR	300/300	12	105	183	9	93	198	0.627	7
		Geleijns et al.	2005	Netherlands	Caucasian	PCR	272/212	3	61	208	1	40	171	0.406	5
Vitiligo	FAS −1377A/G	Li et al.	2008	China	Asian	PCR	750/756	100	378	272	82	346	328	0.514	7
IAA	FAS −1377A/G	Rehman et al.	2018	Pakistan	Caucasian	PCR	170/222	26	23	121	31	39	152	<0.001	7
HT	FAS −1377A/G	Inoue et al.	2016	Japan	Asian	PCR-RFLP	123/87	26	61	36	13	40	34	0.826	6
GD	FAS −1377A/G	Inoue et al.	2016	Japan	Asian	PCR-RFLP	160/87	27	78	55	13	40	34	0.826	6
AIH	FAS −1377A/G	Hiraide et al.	2005	Japan	Asian	PCR	74/98	13	28	33	25	39	34	0.051	4
AA	FAS −1377A/G	Fan et al.	2010	China	Asian	PCR	84/84	12	32	40	7	42	35	0.252	6

### Meta-analysis results of the *FAS* −670 A/G and −1377 G/A polymorphisms and autoimmune diseases

A summary of the meta-analysis of the association between the *FAS* −670 A/G and −1377 G/A polymorphisms and autoimmune diseases is shown in Table 2. In the *FAS* −670 A/G polymorphism, a significant association between *FAS* −670 A/G and the risk of autoimmune diseases was observed under the heterozygous genetic model (GG vs. GA: OR = 1.079, 95% CI 1.004–1.160, *P*=0.038). In the *FAS* −1377 G/A polymorphism, our results indicated that *FAS* −1377 G/A polymorphism was associated with the risk of autoimmune diseases (A vs. G: OR = 1.11, 95% CI = 1.03–1.20, *P*=0.008; AA vs. GG: OR = 1.23, 95% CI = 1.03–1.47, *P*=0.024; AA+AG vs. GG: OR = 1.14, 95% CI = 1.02–1.26, *P*=0.015).

**Table 2:** Meta-analysis for the association between *FAS* −670A/G and −1377G/A polymorphisms and autoimmune diseases stratified by ethnicity, disease type and quality score

Polymorphism	Categories	Studies (*n*)	Test of heterogeneity		Test of associations		Egger’s test	Sensitivity analysis
			*P*-value	*I*^2^ (%)	OR (95% CI)	*P-*value	*P-*value	*P*-value
*FAS* −670 A/G G vs. A								
	Overall	52	<0.001	65.9	0.99 (0.95, 1.03)	0.493	0.222	0.295
	Caucasian	27	<0.001	71.5	1.03 (0.98, 1.08)	0.241	0.973	0.418
	Asian	18	0.225	19.2	0.89 (0.83, 0.96)	0.002	0.147	0.002
	High quality	28	<0.001	70.9	0.98 (0.94, 1.03)	0.446	0.314	0.427
	Low quality	24	<0.001	59.3	1.00 (0.93, 1.08)	0.958	0.622	0.328
	SLE	10	<0.001	73.6	0.85 (0.77, 0.94)	0.001	0.583	<0.001
	RA	6	0.264	22.6	1.04 (0.88, 1.23)	0.675	0.772	0.675
	MS	6	0.313	15.7	0.92 (0.82, 1.03)	0.148	0.826	0.348
	AIH	4	0.003	78.2	0.89 (0.74, 1.08)	0.232	0.089	0.232
	LN	4	0.041	63.6	0.82 (0.62, 1.08)	0.159	0.531	0.201
	SSc	4	0.002	80.2	1.01 (0.95, 1.09)	0.707	0.419	0.707
	AA	3	0.024	73.3	0.93 (0.72, 1.19)	0.553	0.372	0.553
	pSS	3	0.692	0.0	1.02 (0.76, 1.36)	0.914	0.285	0.914
	HT	2	0.082	67.0	1.45 (1.10, 1.90)	0.008	NA	0.008
	GBS	2	0.709	0.0	1.03 (0.87, 1.23)	0.729	NA	0.729
	PBC	2	0.828	0.0	0.82 (0.59, 1.14)	0.240	NA	0.240
*FAS* −670 A/G GG vs. AA								
	Overall	52	<0.001	59.1	0.96 (0.89, 1.04)	0.288	0.104	0.375
	Caucasian	27	<0.001	63.8	1.03 (0.94, 1.14)	0.524	0.519	0.368
	Asian	18	0.225	19.2	0.81 (0.70, 0.94)	0.005	0.196	0.005
	High quality	28	<0.001	62.6	0.95 (0.86, 1.04)	0.244	0.078	0.556
	Low quality	24	<0.001	56.1	0.99 (0.85, 1.16)	0.905	0.287	0.304
	SLE	10	<0.001	73.2	0.74 (0.61, 0.89)	0.002	0.230	<0.001
	RA	6	0.263	22.7	1.05 (0.75, 1.48)	0.762	0.930	0.762
	MS	6	0.353	9.9	0.87 (0.69, 1.10)	0.239	0.686	0.467
	AIH	4	0.004	77.2	0.80 (0.56, 1.15)	0.232	0.123	0.232
	LN	4	0.036	65.0	0.68 (0.39, 1.18)	0.173	0.843	0.226
	SSc	4	0.002	79.1	1.04 (0.91, 1.19)	0.567	0.342	0.567
	AA	3	0.003	82.9	0.68 (0.36, 1.28)	0.235	0.805	0.235
	pSS	3	0.683	0.0	1.00 (0.56, 1.79)	0.998	0.044	0.286
	HT	2	0.062	71.4	2.05 (1.19, 3.54)	0.010	NA	0.010
	GBS	2	0.818	0.0	1.12 (0.79, 1.59)	0.510	NA	0.510
	PBC	2	0.913	0.0	0.69 (0.37, 1.28)	0.234	NA	0.234
*FAS* −670 A/G GG vs. GA								
	Overall	52	0.018	31.4	1.079 (1.004, 1.160)	0.038	0.087	0.006
	Caucasian	27	<0.001	54.4	1.12 (1.03, 1.23)	0.012	0.008	0.001
	Asian	18	0.835	0.0	0.99 (0.86, 1.14)	0.905	0.991	0.905
	High quality	28	0.003	47.3	1.07 (0.99, 1.17)	0.096	0.022	0.028
	Low quality	24	0.454	0.5	1.10 (0.95, 1.27)	0.208	0.364	0.180
	SLE	10	0.568	0.0	0.92 (0.77, 1.11)	0.398	0.177	0.493
	RA	6	0.276	20.9	0.96 (0.69, 1.31)	0.781	0.497	0.781
	MS	6	0.766	0.0	1.05 (0.85, 1.30)	0.674	0.627	0.995
	AIH	4	0.151	43.5	0.90 (0.64, 1.27)	0.563	0.005	0.563
	LN	4	0.910	0.0	0.83 (0.49, 1.39)	0.471	0.142	0.621
	SSc	4	0.243	28.2	1.20 (1.07, 1.36)	0.003	0.201	0.003
	AA	3	0.009	78.7	0.79 (0.44, 1.41)	0.419	0.355	0.419
	pSS	3	0.252	23.9	1.10 (0.66, 1.85)	0.715	0.358	0.071
	HT	2	0.267	18.9	1.52 (0.93, 2.50)	0.098	NA	0.098
	GBS	2	0.726	0.0	1.33 (0.96, 1.85)	0.089	NA	0.089
	PBC	2	0.809	0.0	1.23 (0.71, 2.16)	0.461	NA	0.461
*FAS* −670 A/G GG+GA vs. AA								
	Overall	52	<0.001	70.6	0.94 (0.89, 1.00)	0.051	0.129	0.004
	Caucasian	27	<0.001	76.0	1.00 (0.93, 1.08)	0.945	0.662	0.306
	Asian	18	0.143	26.7	0.83 (0.74, 0.92)	0.001	0.056	0.001
	High quality	28	<0.001	76.7	0.94 (0.88, 1.01)	0.071	0.614	0.023
	Low quality	24	<0.001	60.1	0.95 (0.85, 1.08)	0.445	0.500	0.050
	SLE	10	<0.001	74.3	0.78 (0.67, 0.90)	0.001	0.374	<0.001
	RA	6	0.388	4.4	1.09 (0.85, 1.40)	0.503	0.388	0.503
	MS	6	0.080	49.1	0.83 (0.70, 0.99)	0.043	0.752	0.261
	AIH	4	0.026	67.6	0.87 (0.65, 1.15)	0.330	0.170	0.330
	LN	4	<0.001	84.2	0.86 (0.57, 1.31)	0.483	0.922	0.196
	SSc	4	0.014	71.7	0.92 (0.82, 1.02)	0.112	0.424	0.112
	AA	3	0.009	78.5	0.95 (0.66, 1.39)	0.804	0.666	0.804
	pSS	3	0.741	0.0	0.98 (0.62, 1.54)	0.921	0.964	0.874
	HT	2	0.150	51.7	1.58 (1.03, 2.42)	0.037	NA	0.037
	GBS	2	0.988	0.0	0.92 (0.72, 1.19)	0.536	NA	0.536
	PBC	2	0.976	0.0	0.61 (0.36, 1.03)	0.066	NA	0.066
*FAS* −670 A/G GG vs. GA+AA								
	Overall	52	0.003	38.8	1.04 (0.97, 1.11)	0.294	0.083	0.142
	Caucasian	27	0.001	52.7	1.10 (1.01, 1.19)	0.035	0.175	0.011
	Asian	18	0.694	0.0	0.91 (0.80, 1.04)	0.162	0.541	0.162
	High quality	28	0.005	45.3	1.03 (0.95, 1.12)	0.457	0.019	0.226
	Low quality	24	0.066	32.2	1.06 (0.93, 1.21)	0.421	0.284	0.638
	SLE	10	0.065	44.1	0.86 (0.72, 1.01)	0.071	0.292	0.034
	RA	6	0.237	26.4	0.99 (0.74, 1.34)	0.962	0.627	0.962
	MS	6	0.824	0.0	0.97 (0.80, 1.19)	0.796	0.714	0.709
	AIH	4	0.027	67.3	0.86 (0.63, 1.18)	0.345	0.060	0.345
	LN	4	0.895	0.0	0.71 (0.43, 1.15)	0.162	0.193	0.303
	SSc	4	0.028	67.2	1.14 (1.02, 1.28)	0.022	0.275	0.022
	AA	3	0.009	79.0	0.77 (0.44, 1.34)	0.349	0.546	0.349
	pSS	3	0.338	0.0	1.07 (0.66, 1.75)	0.081	0.426	0.081
	HT	2	0.122	58.1	1.68 (1.06, 2.68)	0.029	NA	0.029
	GBS	2	0.678	0.0	1.24 (0.91, 1.69)	0.172	NA	0.172
	PBC	2	0.787	0.0	0.99 (0.66, 1.75)	0.960	NA	0.960
*FAS* −1377 G/A A vs. G								
	Overall	15	0.091	34.6	1.11 (1.03, 1.20)	0.008	0.329	0.006
	Caucasian	7	0.173	33.4	0.98 (0.82, 1.16)	0.790	0.357	0.863
	Asian	8	0.198	28.8	1.15 (1.05, 1.25)	0.002	0.167	0.002
	High quality	10	0.116	36.5	1.14 (1.05, 1.24)	0.002	0.285	0.001
	Low quality	5	0.293	19.2	0.96 (0.79, 1.18)	0.711	0.588	0.711
*FAS* −1377 G/A AA vs. GG								
	Overall	15	0.452	0.0	1.23 (1.03, 1.47)	0.024	0.878	0.020
	Caucasian	7	0.459	0.0	1.06 (0.67, 1.66)	0.816	0.752	0.881
	Asian	8	0.353	10.0	1.27 (1.04, 1.54)	0.018	0.511	0.018
	High quality	10	0.702	0.0	1.31 (1.08, 1.59)	0.007	0.234	0.005
	Low quality	5	0.325	14.1	0.86 (0.54, 1.38)	0.536	0.072	0.536
*FAS* −1377 G/A AA vs. AG								
	Overall	15	0.863	0.0	1.12 (0.93, 1.34)	0.234	0.584	0.323
	Caucasian	7	0.570	0.0	1.30 (0.76, 2.20)	0.335	0.883	0.680
	Asian	8	0.858	0.0	1.09 (0.90, 1.33)	0.360	0.444	0.360
	High quality	10	0.820	0.0	1.12 (0.92, 1.36)	0.268	0.958	0.375
	Low quality	5	0.507	0.0	1.11 (0.70, 1.77)	0.662	0.166	0.662
*FAS* −1377 G/A AA+AG vs. GG								
								
	Overall	15	0.055	39.9	1.14 (1.02, 1.26)	0.015	0.113	0.008
	Caucasian	7	0.190	31.1	0.95 (0.78, 1.16)	0.620	0.366	0.798
	Asian	8	0.157	34.0	1.21 (1.07, 1.36)	0.002	0.080	0.002
	High quality	10	0.047	47.5	1.17 (1.05, 1.31)	0.005	0.257	0.002
	Low quality	5	0.402	0.7	0.96 (0.74, 1.23)	0.727	0.560	0.727
*FAS* −1377 G/A AA vs. AG+GG								
	Overall	15	0.741	0.0	1.16 (0.98, 1.37)	0.090	0.888	0.097
	Caucasian	7	0.490	0.0	1.10 (0.70, 1.72)	0.674	0.823	0.834
	Asian	8	0.683	0.0	1.17 (0.97, 1.40)	0.098	0.959	0.098
	High quality	10	0.823	0.0	1.20 (0.99, 1.44)	0.054	0.444	0.056
	Low quality	5	0.392	2.5	0.96 (0.63, 1.47)	0.848	0.120	0.848

### Stratification analyses by ethnicity, disease type, and quality score

Based on ethnicity, disease type, and quality score, we performed stratification analyses.

The results of the meta-analysis of the association between the *FAS* −670 A/G and −1377 G/A polymorphisms and autoimmune diseases risk stratified by ethnicity, disease type, and quality score are shown in Table 2.

On the basis of ethnicity, the stratified meta-analysis showed an association between *FAS* −670 A/G polymorphism and the risk of autoimmune diseases in Caucasians (GG vs. GA: OR = 1.12, 95% CI 1.03–1.23, *P*=0.012) and Asians (G vs. A: OR = 0.89, 95% CI 0.83–0.96, *P*=0.002) but not in other ethnic groups. The association between *FAS* −1377 G/A polymorphism and the risk of autoimmune diseases was observed in Asians (A vs. G: OR = 1.15, 95% CI 1.05–1.25, *P*=0.002) but not in Caucasians.

On the basis of disease type, the stratified meta-analysis suggested that the *FAS* −670 A/G polymorphism might be associated with the risk of SLE (G vs. A: OR = 0.85, 95% CI 0.77–0.94, *P*=0.001), MS (GG+GA vs. AA: OR = 0.83, 95% CI 0.70–0.99, *P*=0.043), SSc (GG vs. GA: OR = 1.20, 95% CI 1.07–1.36, *P*=0.003), and HT (G vs. A: OR = 1.45, 95% CI 1.10–1.90, *P*=0.008). However, no association was observed between the *FAS* −670A/G polymorphism and the risk of RA, AIH, AA, pSS, GBS, PBC, or LN. For *FAS* −1377 G/A polymorphism, subgroup analysis was not performed owing to the limited study number.

On the basis of quality score, the stratified meta-analysis suggested that the *FAS* −670 A/G polymorphism might not be associated with autoimmune diseases in high- or low-quality studies. However, the association between *FAS* −1377 G/A polymorphism and the risk of autoimmune diseases was observed in high-quality studies (A vs. G: OR = 1.14, 95% CI 1.05–1.24, *P*=0.002) but not in low-quality studies.

Stratification analysis showed that ethnicity, disease type, and quality score might be the factors of heterogeneity across all studies of association between *FAS* −670 A/G polymorphism and autoimmune diseases risk, and quality score may be the factor of heterogeneity across all studies of association between *FAS* −1377 G/A polymorphism and autoimmune diseases risk.

### Stratification analysis by ethnicity for SLE, RA, MS, AIH, LN, SSc, AA, and pSS

The associations between the *FAS* −670 A/G polymorphism and SLE, RA, MS, AIH, LN, SSc, AA, and pSS are summarized in Table 3 (for *FAS* −1377 G/A polymorphism, subgroup analysis was not performed owing to the limited study number). An association between the *FAS* −670 A/G polymorphism and the risk of autoimmune diseases was observed in Asian patients with SLE (G vs. A: OR = 0.84, 95% CI 0.74–0.95, *P*=0.007) or AIH (G vs. A: OR = 0.55, 95% CI 0.40–0.76, *P*<0.001) and in Caucasian patients with SLE (G vs. A: OR = 0.80, 95% CI 0.67–0.96, *P*=0.015), MS (GG+GA vs. AA: OR = 0.80, 95% CI 0.66–0.96, *P*=0.018), or SSc (GG vs. GA: OR = 1.22, 95% CI 1.07–1.39, *P*=0.003). However, no significant risk was found in any specific ethnicity for RA, LN, AA, or pSS.

**Table 3:** Meta-analysis for the association between *FAS* −670 A/G polymorphism and SLE, RA, MS, AIH, LN, SSc, AA, and pSS stratified by ethnicity

Diseases	FAS −670A/G polymorphism	Population	Studies (*n*)	Test of heterogeneity		Test of associations		Egger’s test *P-*value	Power analysis (%)	Sensitivity analysis value
				*P*-value	*I*^2^ (%)	OR (95% CI)	*P-*value			
SLE										
	G vs. A	Caucasian	4	0.017	70.7	0.80 (0.67, 0.96)	0.015	0.634	73.1	0.015
		Asian	4	0.296	18.9	0.84 (0.74, 0.95)	0.007	0.634	80.2	0.007
	GG vs. AA	Caucasian	4	0.011	73.2	0.68 (0.49, 0.94)	0.021	0.279	63.9	0.021
		Asian	4	0.209	33.9	0.71 (0.55, 0.92)	0.010	0.545	72.9	0.010
	GG vs. GA	Caucasian	4	0.130	46.9	0.96 (0.70, 1.31)	0.797	0.288	5.1	0.797
		Asian	4	0.848	0.0	0.93 (0.72, 1.18)	0.537	0.600	13.7	0.537
	GG+GA vs. AA	Caucasian	4	0.027	67.4	0.70 (0.54, 0.92)	0.011	0.442	81.6	0.011
		Asian	4	0.091	53.6	0.77 (0.63, 0.93)	0.007	0.581	76.7	0.007
	GG vs. GA+AA	Caucasian	4	0.056	60.4	0.84 (0.63, 1.12)	0.228	0.272	19.2	0.228
		Asian	4	0.767	0.0	0.83 (066, 1.05)	0.118	0.551	40.4	0.118
RA										
	G vs. A	Caucasian	5	0.197	33.7	1.06 (0.88, 1.27)	0.520	0.838	5.7	0.149
	GG vs. AA	Caucasian	5	0.169	37.8	1.07 (0.74, 1.55)	0.723	0.956	8.6	0.250
	GG vs. GA	Caucasian	5	0.309	16.6	0.88 (0.62, 1.24)	0.471	0.633	21.4	0.941
	GG+GA vs. AA	Caucasian	5	0.568	0.0	1.19 (0.90, 1.56)	0.221	0.376	27.6	0.103
	GG vs. GA+AA	Caucasian	5	0.178	36.5	0.95 (0.69, 1.32)	0.764	0.737	10.1	0.764
MS										
	G vs. A	Caucasian	5	0.282	20.9	0.90 (0.80, 1.02)	0.095	0.863	23.3	0.242
	GG vs. AA	Caucasian	5	0.296	18.6	0.84 (0.66, 1.08)	0.172	0.981	10.4	0.356
	GG vs. GA	Caucasian	5	0.678	0.0	1.07(0.85, 1.34)	0.576	0.410	17.7	0.899
	GG+GA vs. AA	Caucasian	5	0.103	48.1	0.80 (0.66, 0.96)	0.018	0.754	49.9	0.139
	GG vs. GA+AA	Caucasian	5	0.703	0.0	0.97 (0.79, 1.20)	0.809	0.710	5.0	0.716
LN										
	G vs. A	Asian	2	0.796	0.0	0.79 (0.55, 1.14)	0.201	NA	19.7	0.201
	GG vs. AA	Asian	2	0.634	0.0	0.62 (0.28, 1.35)	0.226	NA	18.7	0.226
	GG vs. GA	Asian	2	0.480	0.0	0.83 (0.40, 1.72)	0.621	NA	6.7	0.621
	GG+GA vs. AA	Asian	2	0.964	0.0	0.69 (0.40, 1.21)	0.196	NA	21.3	0.196
	GG vs. GA+AA	Asian	2	0.528	0.0	0.75 (0.37, 1.49)	0.407	NA	10.3	0.407
SSc										
	G vs. A	Caucasian	2	<0.001	93.3	1.01 (0.94, 1.09)	0.688	NA	6.3	0.688
	GG vs. AA	Caucasian	2	<0.001	92.8	1.05 (0.91, 1.22)	0.476	NA	84.1	0.476
	GG vs. GA	Caucasian	2	0.056	72.6	1.22 (1.07, 1.39)	0.003	NA	10.3	0.003
	GG+GA vs. AA	Caucasian	2	0.001	90.5	0.92 (0.82, 1.03)	0.137	NA	33.6	0.137
	GG vs. GA+AA	Caucasian	2	0.003	88.4	1.15 (1.02, 1.30)	0.021	NA	64.5	0.021
AIH										
	G vs. A	Caucasian	2	0.368	0.0	1.14 (0.91, 1.43)	0.265	NA	13.4	0.265
		Asian	2	0.786	0.0	0.55 (0.40, 0.76)	<0.001	NA	95.9	<0.001
	GG vs. AA	Caucasian	2	0.369	0.0	1.29 (0.82, 2.02)	0.276	NA	5.9	0.276
		Asian	2	0.591	0.0	0.31 (0.16, 0.60)	<0.001	NA	51.7	<0.001
	GG vs. GA	Caucasian	2	0.776	0.0	1.16 (0.76, 1.75)	0.489	NA	13.1	0.489
		Asian	2	0.230	30.7	0.54 (0.29, 1.00)	0.051	NA	95.4	0.051
	GG+GA vs. AA	Caucasian	2	0.369	0.0	1.17 (0.82, 1.68)	0.384	NA	12.6	0.384
		Asian	2	0.658	0.0	0.48 (0.29, 0.79)	0.004	NA	84.2	0.004
	GG vs. GA+AA	Caucasian	2	0.560	0.0	1.20 (0.82, 1.77)	0.350	NA	8.5	0.350
		Asian	2	0.303	5.7	0.44 (0.25, 0.78)	0.005	NA	84.3	0.005
AA										
	G vs. A	Caucasian	2	0.021	81.2	1.06 (0.78, 1.45)	0.703	NA	9.7	0.703
	GG vs. AA	Caucasian	2	0.001	91.1	0.75 (0.32, 1.74)	0.496	NA	25.0	0.496
	GG vs. GA	Caucasian	2	0.002	89.3	0.50 (0.22, 1.10)	0.086	NA	5.2	0.086
	GG+GA vs. AA	Caucasian	2	0.118	59.2	1.39 (0.87, 2.23)	0.172	NA	34.0	0.172
	GG vs. GA+AA	Caucasian	2	0.001	90.1	0.61 (0.29, 1.32)	0.211	NA	15.2	0.211
pSS										
	G vs. A	Caucasian	2	0.096	64.0	1.19 (0.88, 1.60)	0.252	NA	20.7	0.252
	GG vs. AA	Caucasian	2	0.097	63.7	1.40 (0.77, 2.55)	0.273	NA	32.5	0.273
	GG vs. GA	Caucasian	2	0.015	83.0	1.49 (0.87, 2.56)	0.144	NA	24.4	0.144
	GG+GA vs. AA	Caucasian	2	0.783	0.0	1.10 (0.69, 1.74)	0.694	NA	6.8	0.694
	GG vs. GA+AA	Caucasian	2	0.020	81.7	1.49 (0.89, 2.47)	0.128	NA	34.0	0.128

Abbreviation: NA, not available.

### Publication bias

The Egger’s test was performed to assess the publication bias under all genetic models of the meta-analysis and the results are shown in Table 2. For the *FAS* −670 A/G polymorphism, the results from Egger’s tests indicated evidence for publication bias in the homozygous model for pSS, heterozygous models for Caucasians, AIH and high-quality studies, and recessive models for high-quality studies (*P*=0.044, 0.008, 0.005, 0.022, and 0.019, respectively). After adjustment by the trim-and-fill method, the ORs corrected for publication bias were not qualitatively different for the five models (OR = 1.30, 95% CI = 0.79–2.13, *P*=0.298; OR = 1.13, 95% CI = 1.03–1.23, *P*=0.007; OR = 0.92, 95% CI = 0.65–1.30, *P*=0.636; OR = 1.09, 95% CI = 1.00–1.18, *P*=0.052; and OR = 1.04, 95% CI = 0.96–1.13, *P*=0.323, respectively). No publication bias was found among the studies regarding the association between *Fas* −1377 G/A polymorphism and autoimmune diseases risk (all *P*>0.05). Therefore, the presence of publication bias did not influence the stability of the results. In addition, the results concerning association between *FAS* −670 A/G polymorphism and SLE, RA, MS, AIH, LN, SSc, AA, and pSS stratified by ethnicity did not show any evidence of publication bias (Table 3).

### Sensitivity analysis

The genotype frequencies in the controls of five articles [22,31,51,52,55] deviated significantly from the HWE, which could cause potential bias. To check the robustness of our results, sensitivity analysis was performed by excluding these five HWE-deviating studies. The corresponding results of the sensitivity analysis are provided in Tables 2 and 3. The results showed that the overall OR changed only under the dominant model (*P*=0.051 vs. 0.004) after excluding the HWE-deviating studies, but the association between the *FAS* −670 A/G polymorphism and autoimmune diseases risk was not qualitatively altered under the heterozygous model (*P*=0.038 vs. 0.006), illustrating that the meta-analysis results were stable. In the stratification analysis by ethnicity, the results in Caucasians and Asians did not change when the HWE-deviating studies were excluded. In the stratification analysis by disease type, the OR changed only under the recessive model (*P*=0.071 vs. 0.034) after excluding the HWE-deviating studies from the analysis of SLE, but the association between the *FAS* −670 A/G polymorphism and SLE risk was not qualitatively altered under the allelic model (*P*=0.001 vs. <0.001). However, the association between *FAS* −670 A/G and MS risk was materially altered under the dominant model (*P*=0.043 vs. 0.261) after excluding the HWE-deviating studies. Similarly, a change was observed in the analysis of Caucasian patients with MS under the dominant model (*P*=0.018 vs. 0.139). In the stratification analysis by quality score, the association between *FAS* −670 A/G and high-quality studies was materially altered under the heterozygous and dominant model (*P*=0.096 vs. 0.028; *P*=0.071 vs. 0.023) after excluding the HWE-deviating studies. Additionally, the results of the association between *FAS* −1377 G/A and autoimmune diseases risk did not change when the HWE-deviating studies were excluded in five models.

### FPRP analysis results

The FPRP values were calculated for the main significant associations and the results are shown in Table 4. For a prior probability of 0.1, the FPRP values indicated that four genetic models (*FAS* −670 A/G: GG vs. GA; *FAS* −1377 G/A: A vs. G; *FAS* −1377 G/A: AA vs. GG; *FAS* −1377 G/A: AA+AG vs. GG) of the *FAS* −670 A/G and −1377 G/A polymorphisms were truly associated with an increased risk of autoimmune diseases (FPRP = 0.262, 0.073, 0.173, and 0.085, respectively). Furthermore, with regard to the *FAS* −670 A/G polymorphism, noteworthy results were found in Asians, Caucasians, SLE, HT, SSc, and MS. Regarding the *FAS* −1377 G/A polymorphism, a positive association was observed in Asians and high-quality studies.

**Table 4:** FPRP values for associations between *FAS* −670A/G and −1377G/A polymorphisms and autoimmune disease PRISMA 2009 Checklist

Genotype	Population	Studies (*n*)	OR (95% CI)	*P*-value^1^	Statistical power^2^	Prior probability				
						0.25	0.1	0.01	0.001	0.0001
FAS −670 A/G G vs. A										
	Asian	18	0.89 (0.83, 0.96)	0.003	1.000	0.008^3^	0.022^3^	0.202^3^	0.718	0.962
	SLE	10	0.85 (0.77, 0.94)	0.002	1.000	0.005^3^	0.014^3^	0.133^3^	0.608	0.939
	HT	2	1.45 (1.10, 1.90)	0.007	0.597	0.034^3^	0.096^3^	0.539	0.922	0.992
FAS −670 A/G GG vs. AA										
	Asian	18	0.81 (0.70, 0.94)	0.006	0.995	0.016^3^	0.048^3^	0.355^3^	0.847	0.982
	SLE	10	0.74 (0.61, 0.89)	0.001	0.866	0.005^3^	0.014^3^	0.137^3^	0.615	0.941
	HT	2	2.05 (1.19, 3.54)	0.010	0.131	0.186^3^	0.407^3^	0.883	0.987	0.999
FAS −670 A/G GG vs. GA										
	Overall	52	1.079 (1.004, 1.160)	0.040	1.000	0.106^3^	0.262^3^	0.796	0.975	0.997
	Caucasian	27	1.12 (1.03, 1.23)	0.018	1.000	0.051^3^	0.138^3^	0.637	0.947	0.994
	SSc	4	1.20 (1.07, 1.36)	0.004	1.000	0.013^3^	0.037^3^	0.299^3^	0.811	0.977
FAS −670 A/G GG+GA vs. AA										
	Asian	18	0.83 (0.74, 0.92)	<0.001	1.000	0.001^3^	0.003^3^	0.037^3^	0.280^3^	0.795
	SLE	10	0.78 (0.67, 0.90)	<0.001	0.984	0.002^3^	0.006^3^	0.063^3^	0.403^3^	0.871
	MS	6	0.83 (0.70, 0.99)	0.038	0.993	0.104^3^	0.258^3^	0.792	0.975	0.997
	HT	2	1.58 (1.03, 2.42)	0.035	0.406	0.208^3^	0.440^3^	0.896	0.989	0.999
FAS −670 A/G GG vs. GA+AA										
	Caucasian	27	1.10 (1.01, 1.19)	0.018	1.000	0.050^3^	0.136^3^	0.634	0.946	0.994
	SSc	4	1.14 (1.02, 1.28)	0.027	1.000	0.074^3^	0.193^3^	0.725	0.964	0.996
	HT	2	1.68 (1.06, 2.68)	0.029	0.317	0.218^3^	0.455^3^	0.902	0.989	0.999
FAS −1377 G/A A vs. G										
	Overall	15	1.11 (1.03, 1.20)	0.009	1.000	0.025^3^	0.073^3^	0.464^3^	0.897	0.989
	Asian	8	1.15 (1.05, 1.25)	0.001	1.000	0.003^3^	0.009^3^	0.092^3^	0.504	0.911
	High quality	10	1.14 (1.05, 1.24)	0.002	1.000	0.007^3^	0.020^3^	0.183^3^	0.693	0.958
FAS −1377 G/A AA vs. GG										
	Overall	15	1.23 (1.03, 1.47)	0.023	0.985	0.065^3^	0.173^3^	0.696	0.959	0.996
	Asian	8	1.27 (1.04, 1.54)	0.015	0.955	0.045^3^	0.125^3^	0.610	0.940	0.994
	High quality	10	1.31 (1.08, 1.59)	0.007	0.915	0.020^3^	0.058^3^	0.405^3^	0.873	0.986
FAS −1377 G/A AA+AG vs. GG										
	Overall	15	1.14 (1.02, 1.26)	0.010	1.000	0.030^3^	0.085^3^	0.505	0.911	0.990
	Asian	8	1.21 (1.07, 1.36)	0.001	1.000	0.004^3^	0.012^3^	0.121^3^	0.581	0.933
	High quality	10	1.17 (1.05, 1.31)	0.005	1.000	0.019^3^	0.055^3^	0.391^3^	0.866	0.985

1 Chi-square test was used to calculate the genotype frequency distributions.

2 Statistical power was calculated using the number of observations in the subgroup and the OR and *P*-values in this table.

3 The level of FPRP threshold was set at 0.5 and noteworthy findings are presented.

### TSA results

**Figure 2:**
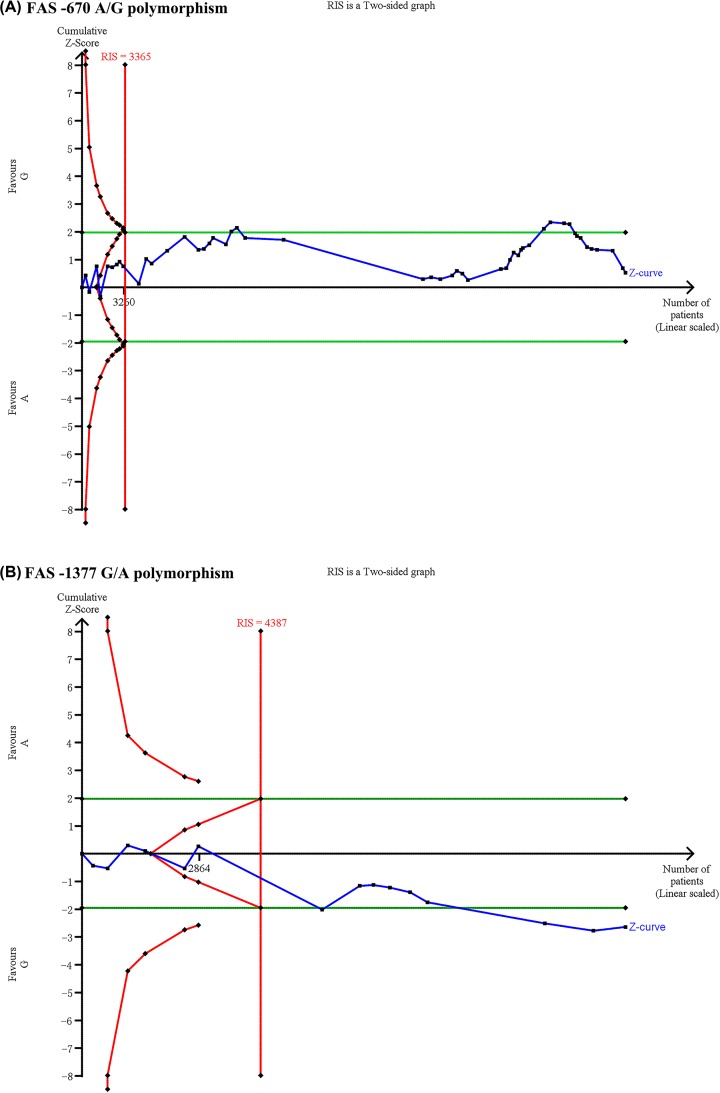
Trial sequential analyses of the associations between *FAS* polymorphisms and autoimmune diseases risk The RIS was calculated based on a type I error = 5%, power = 80%, and RRR = 20%. (**A**) *FAS* −670 A/G polymorphism; (**B**) *FAS* −1377G/A polymorphism.

In the TSA of association of *FAS* −670 A/G polymorphism and autoimmune diseases risk, the cumulative Z-curve neither crossed conventional boundary nor trial sequential monitoring boundary, however, the sample size reached RIS (3365) in allelic model (Figure 2A). In the TSA of association of *FAS* −1377 G/A polymorphism and autoimmune diseases risk, the sample size also reached RIS (4387) and the cumulative Z-curve crossed the conventional boundary, although the cumulative Z-curve did not cross trial sequential monitoring boundary in allelic model (Figure 2B). The TSA results indicated that the cumulative evidence was reliable and sufficient, and no additional studies were required.

## Discussion

Our results showed that ethnicity, disease type, and quality score may be the factors of heterogeneity across all studies of association between *FAS* −670 A/G polymorphism and autoimmune diseases, and quality score may be the factor of heterogeneity across all studies of association between *FAS* −1377 G/A polymorphism and autoimmune diseases. In the ethnicity stratification analysis, the results of our meta-analysis revealed diverse associations between the *FAS* −670 A/G and −1377 G/A polymorphisms and various autoimmune diseases in different ethnic groups. The findings indicated that the *FAS* gene polymorphisms might play different roles in different ethnic groups. This suggests that ethnic differences may be involved in the genetic backgrounds of these patients. There are several possible explanations for such an ethnic discrepancy. First, different populations usually have different patterns of linkage disequilibrium. The *FAS* −670 A/G and −1377 G/A polymorphisms may be in close linkage with different nearby causal variants in different populations. Second, the *FAS* −670 A/G and −1377 G/A polymorphisms may interact with environmental and genetic factors or combined effects among different ethnicities. Furthermore, lifestyle factors such as alcohol consumption, cigarette smoking, nutritional status, and menopausal status may also explain this discrepancy. Finally, study numbers and sample sizes were relatively small in the stratification analysis by ethnicity, which may have resulted in inadequate statistical power to detect associations between the *FAS* −670 A/G and −1377 G/A polymorphisms and autoimmune diseases.

In the disease-type stratification analysis, the *FAS* −670 G allele was associated with an increased risk of SSc and HT and with a decreased risk of SLE, MS, and AIH (in Asians) but was not associated with other autoimmune diseases. These findings may reflect differences in the risks of various autoimmune diseases due to differences in environmental and genetic backgrounds. The present results indicate that the *FAS* −670 G allele is associated with a decreased risk of SLE, MS, and AIH (in Asians), which conflicts with a previous finding that the *FAS* −670 G allele in the *FAS* promoter was associated with an increased risk of autoimmune diseases [22,78]. One possible mechanism by which this allele may reduce the risk of SLE, MS, and AIH (in Asians) is by a reduction in soluble FAS (sFAS). The FAS protein exists in two isoforms, one a transmembrane protein and the other a soluble protein. sFAS expression is highly regulated at the mRNA transcript level [79,80]. Transcription of both FAS and sFAS is driven by the same gene promoter [22], with alternative splicing of the *FAS* mRNA resulting in a variant that lacks exon 6, which encodes the transmembrane domain of FAS [81]. Plasma sFAS, an antiapoptotic molecule, has been found to block apoptosis in autoreactive lymphocytes by competing with FAS for FASL or soluble FASL binding in SLE, MS, and AIH (in Asians) [79,82–85]. Similarly, this may explain why the *FAS* −670 G allele was associated with an increased risk of autoimmune diseases in Caucasians and with a decreased risk in Asians. For *FAS* −1377 G/A polymorphism, subgroup analysis was not performed owing to the limited study number. The *FAS* −1377 G/A polymorphism occurs at the consensus sequence of transcription factor SP1 binding site in the silencer region [48]. The *FAS* −1377 A allele may destroy SP1 transcription factor binding sites, resulting in reduced promoter activity and FAS expression [18]. Abnormal apoptosis mediated by the FASL interaction with the FAS receptor is involved in the pathogenesis of several autoimmune diseases [19].

We performed a meta-analysis of data from patients diagnosed with autoimmune diseases (SLE, MS, RA, AIH, LN, SSc, AA, pSS, HT, GBS, PBC, vitiligo, GD, T1D, IAA, JIA, and SPA) and healthy controls. This meta-analysis differs from the seven previous meta-analyses [43,61–66] because the present study included 33 more studies (consisting of new studies with same and different disease types) [15,17,22,27–30,32–35,37,41,43–45,50,52–55,59,60] and yielded several novel and distinct findings. One previous meta-analysis [62] including SLE, RA, SSc, pSS, JIA, and SPA demonstrated that the *FAS* −670 A/G polymorphism might be associated with the risk of rheumatic disease, especially in Asians, SLE and RA, and the *FAS* −1377 G/A polymorphism was associated with SLE risk. Compared with this meta-analysis, our meta-analysis focused on overall autoimmune diseases risk and showed that *FAS* −670 A/G polymorphism was associated with autoimmune diseases risk in Caucasians, MS, SSc and HT; and the *FAS* −1377 G/A polymorphism was associated with autoimmune diseases risk in Asians and high quality studies, which were different from the previous meta-analyses. One meta-analysis [43] showed that the *FAS* −670 A/G polymorphism may be associated with SLE risk in the Chinese population. Two meta-analyses [64,66] suggested that the *FAS* −670 A/G and −1377 G/A polymorphisms was associated with the risk of SLE, stratification by ethnicity indicated an association between the *FAS* −670 A/G and SLE in Asian populations. Two meta-analyses [61,63] showed that the *FAS* −670 A/G polymorphism was not associated with the risk of RA. One meta-analysis [65] suggested that the *FAS* −670 A/G polymorphism was not associated with the risk of AIH. These six meta-analyses focused on the association between *FAS* polymorphism and a single disease (SLE, RA, or AIH). Compared with these meta-analyses, our meta-analysis covered overall autoimmune diseases, and subgroup analyses were performed by ethnicity, disease type, and quality score, thereby yielding several novel and distinct findings. Furthermore, some previous meta-analyses [63,65] including several studies [25,30,31,40] made some errors when extracting the data. Thus, we here added 33 new studies [15,17,22,27–30,32–35,37,41,43–45,50,52–55,59,60] on SLE, MS, pSS, AA, PBC, HT, GBS, LN, vitiligo, T1D, IAA, and GD and corrected the previous errors, providing more reliable results. In addition, FPRP test was performed to support that the evidence of our results was robust and sufficiently conclusive, and the result of TSA showed that there was sufficient evidence and much larger sample size to support these conclusions, thereby increasing the statistical power. We strongly believe that our findings can help resolve many of the controversies of the association of *FAS* polymorphism and autoimmune diseases.

Sensitivity analysis are generally performed to assess the robustness of meta-analyses by excluding and including HWE-deviating studies from genetic association studies, which is a recommended approach [86]. Probable explanations for deviation from HWE include nonrandom mating, population stratification, selection bias, genotyping error, inbreeding, genetic drift, chance, differential survival of marker carriers, or combinations of these reasons [87]. However, key empirical evidence does not support a strong association between estimates of genetic effect and deviations from HWE [88]. Nonetheless, the findings of our meta-analysis should be interpreted with caution in the case of material alterations in results after excluding the HWE-deviating studies.

The present study has several limitations that should be considered when interpreting the conclusions. First, only case–control studies were considered for inclusion. Selection bias and unmeasured confounding can occur at both the design and analysis stages of observational studies. Second, this analysis only included articles published in English and Chinese; this may reduce the credibility of the results because of language bias [89]. Third, our study only analyzed a single locus, single nucleotide polymorphism (SNP) −670 A/G and −1377 G/A in the *FAS* gene and did not investigate associations between genetic haplotypes containing the *FAS* −670 A/G and −1377 G/A polymorphisms and the risk of autoimmune diseases because of inadequate haplotype data. It is unknown whether other genetic mutations contribute to changes in the expression or function of the *FAS* gene. For uncovering the genetic causes of disease, haplotypes provide more information and have a greater influence than genotypes and single SNPs. Fourth, most studies included in our analysis were performed in the Caucasian and Asian populations; therefore, our results may apply only to these ethnic groups. Additional studies of other ethnicities are needed. Fifth, autoimmune diseases are multifactorial diseases caused by interactions between genetic and environmental factors, meaning that the *FAS* −670 A/G and −1377 G/A polymorphisms may only partially influence the pathogenesis of autoimmune diseases; this may lead to bias in the present results. Finally, the findings of our meta-analysis should be interpreted with caution in the case of heterogeneity observed under some genetic models.

Translating information of genetic associations into clinical diagnostics would help with improved understanding of the autoimmune diseases’ etiology. Establishing evidence-based medical evidence of genetic susceptibility to autoimmune diseases risk might facilitate the preventive and therapeutic strategies, which has a beneficial clinical utility for not only clinicians and researchers but also patients.

In summary, our meta-analysis suggested that the *FAS* −670 A/G polymorphism might be associated with the risk of autoimmune diseases, especially in Caucasians and Asians, SLE, MS, SSc, and HT. Moreover, the *FAS* −670 A/G polymorphism might be associated with the risk of autoimmune diseases in Asian patients with SLE or AIH and Caucasian patients with SLE, MS, or SSc. The *FAS* −1377 G/A/ polymorphism might be associated with the risk of autoimmune diseases, specifically for Asians and high quality studies. Stratification analysis showed that ethnicity, disease type and quality score might be the factors of heterogeneity across all studies of association between *FAS* −670 A/G polymorphism and autoimmune diseases risk, and quality score might be the factor of heterogeneity across all studies of association between *FAS* −1377 G/A polymorphism and autoimmune diseases risk.

## Abbreviations

AA: alopecia areata
AIH: autoimmune hepatitis
CI: confidence interval
FASL: FAS-ligand
FPRP: false-positive report probability
GBS: Guillain–Barré syndrome
GD: Graves’ disease
HT: Hashimoto’s thyroiditis
HWE: Hardy–Weinberg equilibrium
IAA: idiopathic aplastic anemia
JIA: juvenile idiopathic arthritis
LN: lupus nephritis
MS: multiple sclerosis
NOS: Newcastle–Ottawa scale
OR: odds ratio
PBC: primary biliary cirrhosis
pSS: primary Sjögren’s syndrome
RA: rheumatoid arthritis
RIS: required information size
sFAS: soluble FAS
SLE: systemic lupus erythematosus
SNP: single nucleotide polymorphism
SPA: spondyloarthropathy
SP1: stimulatory protein 1
SSc: systemic sclerosis
TNF: tumor necrosis factor
TSA: trial sequential analysis
T1D: type 1 diabetes mellitus
